# Hyaluronic Acid Loaded with Cerium Oxide Nanoparticles as Antioxidant in Hydrogen Peroxide Induced Chondrocytes Injury: An In Vitro Osteoarthritis Model

**DOI:** 10.3390/molecules25194407

**Published:** 2020-09-25

**Authors:** Yi-Wen Lin, Chih-Hsiang Fang, Fan-Qi Meng, Cherng-Jyh Ke, Feng-Huei Lin

**Affiliations:** 1 Institute of Biomedical Engineering, College of Medicine and College of Engineering, National Taiwan University, No. 1, Sec. 4, Roosevelt Rd, Taipei 10617, Taiwan; zhew520@gmail.com (Y.-W.L.); danny07291991@hotmail.com (C.-H.F.); danny07291991@yahoo.com.tw (F.-Q.M.); 2 Biomaterials Translational Research Center, China Medical University Hospital, No. 2, Yude Rd., North Dist., Taichung City 404332, Taiwan; 3 Division of Biomedical Engineering and Nanomedicine Research, National Health Research Institutes, No. 35, Keyan Road, Zhunan, Miaoli County 35053, Taiwan

**Keywords:** chondrocytes, cerium oxide nanoparticles, oxidative stress, antioxidant, osteoarthritis

## Abstract

Osteoarthritis (OA) is the most common joint disease type and is accompanied by varying degrees of functional limitation. Both hyaluronic acid (HA) joint injections and pain relievers are efficient treatments for early-stage osteoarthritis. However, for the decomposition by hyaluronidase and free radicals in the knee joint, HA injection treatment has limited effect time. The cerium oxide nanoparticles (CeO_2_) is a long time free radical scavenger. CeO_2_ combined with HA expected, may extend the HA decomposition time and have a positive effect on osteoarthritis therapy. In this study, CeO_2_ was successfully synthesized using the hydrothermal method with a particle size of about 120 nm, which possessed excellent dispersibility in the culture medium. The in vitro OA model was established by cell treated with H_2_O_2_ for 30 min. Our study found that the inhibition of chondrocyte proliferation dose-dependently increased with H_2_O_2_ concentration but was significantly decreased by supplementation of cerium oxide nanoparticles. COL2a1 and ACAN gene expression in chondrocytes was significantly decreased after H_2_O_2_ treatment; however, the tendency was changed after cerium oxide nanoparticles treatment, which suggested that damaged chondrocytes were protected against oxidative stress. These findings suggest that cerium oxide nanoparticles are potential therapeutic applications in the early stage of OA.

## 1. Introduction

Osteoarthritis (OA), the most common form of arthritis, has long been considered a complex metabolic disease disorder which leads to focal damage to articular cartilage at the weight-bearing areas [1]. This slowly progressive, disabling joint disorder can significantly impair life quality (QOL) and affects nearly 34% of those ages 65 and older [2,3,4]. Initially, increased pressure on the joint, which leads to the cartilage matrix’s fragility, was considered to be the primary pathological process. As a result of the progress in molecular biology in the 1990s, scientists discovered that many soluble mediators could increase the synthesis of matrix metalloproteinases by chondrocytes and led to the inflammatory process. Recent data have shown that OA is a much more complex metabolic syndrome induced by the inflammatory mediators released by cartilage, subchondral bone, and synovium [5].

The pharmacologic treatment for OA, including nonsteroidal anti-inflammatory drugs, and intraarticular injection of glucocorticoids, is mainly symptomatic; none of these have been shown to detain pathology progression or reverse cartilage damage in patients [6]. Surgery is considered to be the last resort management option for patients who fail to benefit from the more conservative treatment options. Simultaneously, plenty of possible surgery-associated problems such as infection, thromboembolism, as well as nerve and vascular injuries can be complicated with a high risk of mortality in the elderly [7].

Approved by the U.S. Food and Drug Administration, in 1999, as a medical device, intra-articular injection of hyaluronic acid (HA) has recently become one of the favorite non-operative options for the treatment of OA symptoms [8]. HA, a critical constituent of the healthy synovial fluid, increases the synovial fluid’s viscosity and acts as a shock-absorbent to protect soft tissue from trauma. Furthermore, HA facilitates gliding via layer formation on the cartilage, soothes the pain, and is a significant contributor to joints’ homeostasis by exerting immunomodulatory effects on inflammatory cells. The production and activity of pro-inflammatory mediators and matrix metalloproteinases could be reduced [9,10].

When exposed to appropriate stimuli, a burst of oxidative metabolism ensues within polymorphonuclear leukocytes (PMNs) and is accompanied by the generation of superoxid [11,12,13]. Superoxide, a highly reactive free radical, can react with other moieties and generate other oxygen-derived free radicals in aqueous media; these reactive chemical entities can decompose hyaluronic acid and reduce the viscosity of its solutions [14], degrade cartilage proteoglycan [15], and inhibit the normal gelation of soluble collagen [16]. Therefore, reducing free radicals is essential to retard the degradation of hyaluronic acid and protect the cartilage in early OA.

Cerium oxide (CeO_2_) can co-exist and flip-flop between trivalent (+3) and tetravalent (+4) states in a redox reaction; it is considered to be a potent free radical scavenger due to this strong redox capacity [17,18]. Alterations in this oxidation state caused by oxygen vacancies or deficiencies in the lattice structure are dynamic. They can occur spontaneously or in response to different physiological environments [19] or physical parameters [17] as more oxygen vacancies are generated when size decrease. Size is one of the critical parameters in this redox reaction [20]. This suggests that CeO_2_ nanoparticles could emerge as a potent antioxidant agent. Indeed, CeO_2_ nanoparticles have been used as potential therapeutic agents in various oxidative stress diseases/disorders, such as Alzheimer’s disease [21,22,23,24]. In this study, hydrogen peroxide (H_2_O_2_) was used as the source of the free radicals to induce chondrocytes’ injury in an early stage model of OA. The cerium oxide nanoparticles were added into hyaluronic acid; we believe that cerium oxide is capable of protecting hyaluronic acid from degradation, and cerium oxide nanoparticles-loaded hyaluronic acid can inhibit oxidative stress induced by hydrogen peroxide on chondrocytes.

## 2. Results

### 2.1. Morphology and Composition of CeO_2_ Nanoparticles

**Figure 1:**
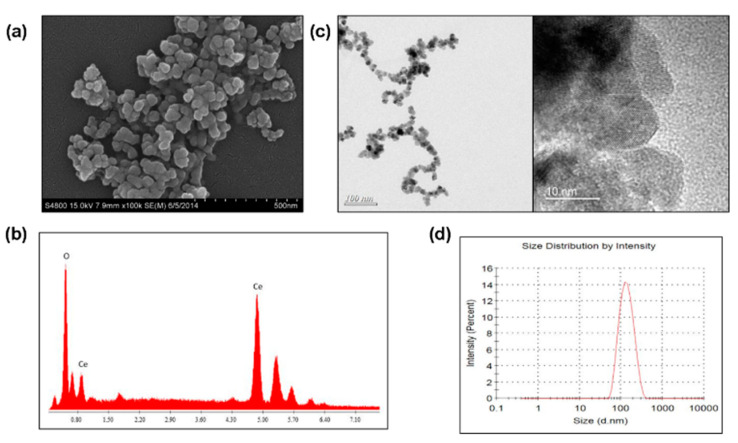
Morphology and composition of CeO_2_ nanoparticles. The morphology and composition of CeO_2_ nanoparticles. (**a**) The surface topography of cerium oxide nanoparticles; (**b**) The EDXA pattern of cerium oxide nanoparticles; (**c**) The TEM image of cerium oxide nanoparticles; (**d**) Particle size distribution.

The morphology and composition of CeO_2_ nanoparticles were analyzed. The SEM images showed that cubic crystals cerium oxide nanoparticles agglomerated, with a particle size from 30 nm to 60 nm (Figure 1a). The chemical composition of the synthesized cerium oxide nanoparticles was analyzed by SEM-accessorized EDS, which indicated the composition was mainly cerium and oxygen (Figure 1b). The TEM images showed that the grain size of cerium oxide nanoparticle was around 10 nm, and the exposing surface was unstable, where three crystal planes could be found by the lattice spacing, namely (111) (0.3127 nm), (100) (0.2711 nm), and (110) (0.1971 nm). (Figure 1c). The particle size distribution of cerium oxide nanoparticles in cell culture medium was 131.1 ± 0.7 nm (PDI = 0.104), which was measured by Zetasizer (Figure 1d).

### 2.2. Crystal Phase Identification

**Figure 2:**
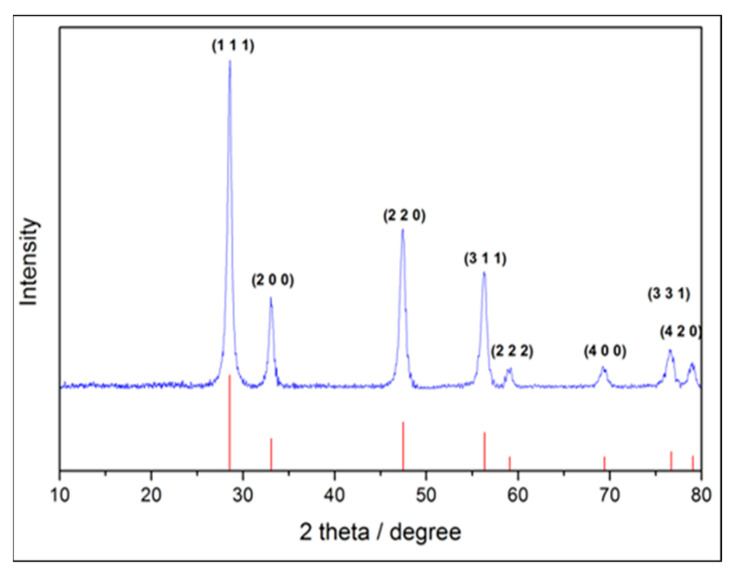
The XRD pattern of CeO_2_ nanoparticles. The characteristic diffraction peaks on 2 theta of (111), (200), (220), (311), (222), (400), (331), and (420), respectively, were fully matched to standard cerium oxide pattern of JCPDS No. 340394.

CeO_2_ nanoparticles were scanned from 10–80°, after drying, polishing, and evenly spreading on XRD sample stage. Figure 2 shows the X-ray pattern of the synthesized cerium oxide nanoparticles; where the characteristic diffraction peaks on 2 theta of (111), (200), (220), (311), (222), (400), (331), and (420), respectively, are fully matched to standard cerium oxide pattern of JCPDS No. 340394. The crystal structure was identified as cerium oxide without the second phase to be traced in the pattern. From the results, we could tell that the crystal structure of synthesized cerium oxide nanoparticles was the same as that of CeO_2_ prepared by a conventional method.

### 2.3. Determination of the Effect of Oxidative Stress on Chondrocytes

**Figure 3:**
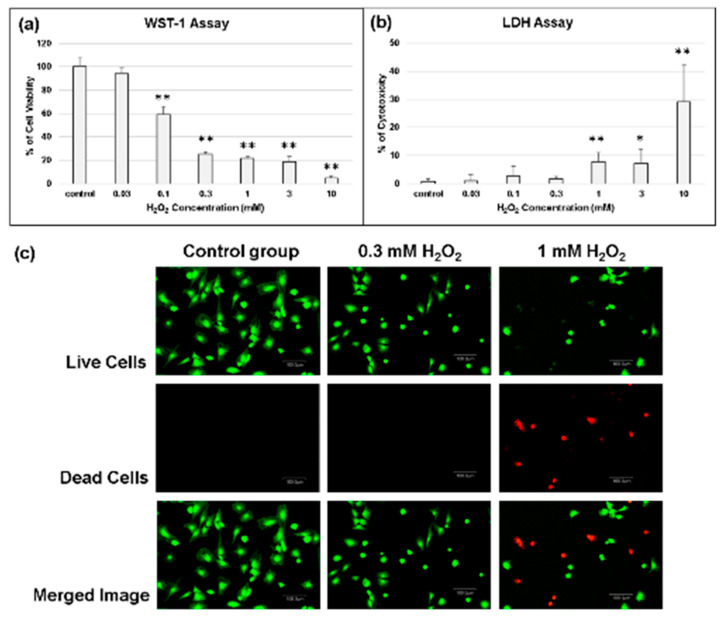
Determination of chondrocytes viability under oxidative stress. (**a**) Water-soluble tetrazolium (WST-1) assay; (**b**) Lactate dehydrogenase (LDH) assay; (**c**) Live/dead staining of chondrocytes under different concentration of H_2_O_2_ treatment for 30 min. * *p* < 0.05 and ** *p* < 0.01 when compared with th control group.

**Figure 4:**
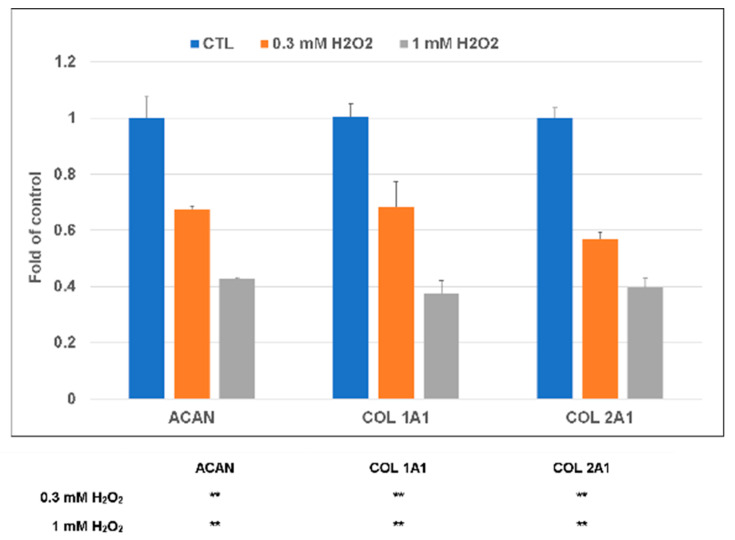
Gene expression of chondrocytes under oxidative stress in the presence of H_2_O_2_ oxidative stress, the expression of COL1A1, COL2A1, and aggrecan (ACAN) genes were downregulated. ** *p* < 0.01 when compared with control group.

In the presence of 0.3 mM H_2_O_2_ oxidative stress for 30 min, there was no significant cytotoxicity, but chondrocytes’ viability was significantly affected (Figure 3a,b). The results of live/dead staining also showed that at the concentration of 0.3 mM H_2_O_2_, the treated chondrocytes did not show any significant evidence of cell death or cell damage, whereas the slightly excessive amount of H_2_O_2_ (1 mM) was harmful to the chondrocytes (Figure 3c). In a further study, 0.3 mM H_2_O_2_ was selected to be the concentration as the inductive agent for the chondrocytes apoptosis [25]. Similar to above, the expression of COL1A1, COL2A1, and aggrecan (ACAN) genes was downregulated in the presence of H_2_O_2_ oxidative stress; this phenomenon was even more evident at 1.0 mM H_2_O_2_ oxidative stress (Figure 4).

### 2.4. Biocompatibility of Cerium Oxide Nanoparticles

**Figure 5:**
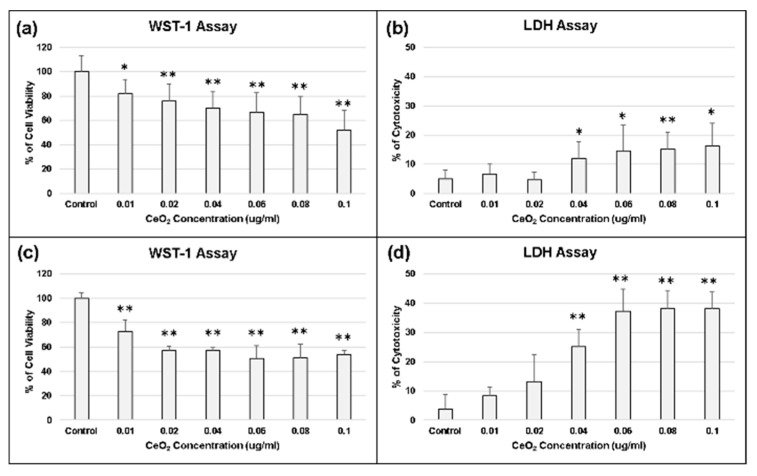
Biocompatibility of CeO_2_ nanoparticles. (**a**) WST-1 assay on day 1; (**b**) LDH assay on day 1; (**c**) WST-1 assay on day 2; (**d**) LDH assay on day 2. At the lower concentration of CeO2 (less than 0.02 μg/mL), it showed no cytotoxic effect and was more biocompatible to chondrocytes. * *p* < 0.05 and ** *p* < 0.01 when compared with control group.

Our results indicated that the viability of chondrocytes was inhibited and their cytotoxicity getting increased as the concentration of CeO_2_ nanoparticles increased (Figure 5). At the lower concentration of CeO_2_ (less than 0.02 μg/mL), it showed no cytotoxic effect and was more biocompatible to chondrocytes (Figure 5).

### 2.5. Cell Apoptosis and Gene Assay

**Figure 6:**
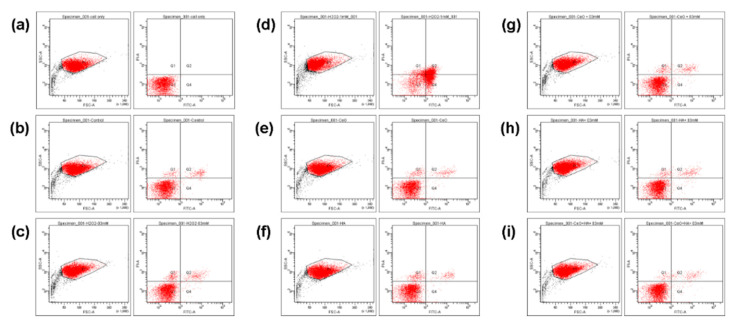
The representative results of cell apoptosis assay by flow cytometry. (**a**) Normal unstained cell for gating; (**b**) Control group; (**c**) 0.3 mM H_2_O_2_; (**d**) 1 mM H_2_O_2_; (**e**) CeO_2_ biocompatibility assay; (**f**) hyaluronic acid (HA) biocompatibility assay; (**g**) CeO_2_ protection; (**h**) HA protection; (**i**) CeO_2_/HA protection. The combination of 0.01 ug/mL CeO_2_ nanoparticles/1% HA could protect chondrocytes from the harmful effect induced by 0.3 mM H_2_O_2_.

In flow cytometry, the results for detection of cell apoptosis rate by Annexin V/PI apoptosis assay are divided into the following four quadrants: Annexin V-/PI-(Q3) which represent living cell, Annexin V+/PI-(Q4) which represent early apoptotic cell, Annexin V+/PI+(Q2) which characterize late stage apoptotic cells, and Annexin V-/PI+(Q1) which identify cells with permeabilized membranes only. As unstained controls, normal cell was used to gate for a negative cell population. The percentage of each quadrant of figures of flow cytometry are summarized in Table 1. The results indicated that 0.3 mM H_2_O_2_ could permeate chondrocytes membranes as that induced in chondrocytes of early OA, 0.02 ng/mL CeO_2_ nanoparticles and 1% HA were biocompatible, and the combination of 0.02 ng/mL CeO_2_ nanoparticles/1% HA could protect chondrocytes from the harmful effect induced by 0.3 mM H_2_O_2_ (Figure 6).

### 2.6. Effect of CeO_2_ Nanoparticles-Loaded Hyaluronic Acid Treatment When Chondrocytes under Oxidative Stress

**Figure 7:**
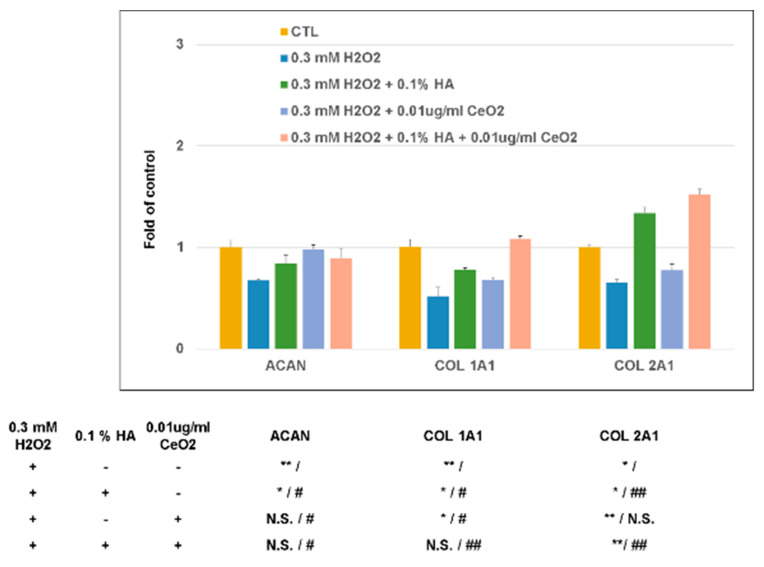
Effect of CeO_2_ nanoparticles-loaded hyaluronic acid treatment when chondrocytes under oxidative stress. The presence of HA and CeO_2_ can scavenger H_2_O_2_ oxidative stress on chondrocytes, while the presence of HA + CeO_2_ can further enhance the scavenger effect of HA on the H_2_O_2_ oxidative stress induced COL1A1 and COL2A1 gene expression on chondrocytes. * *p* < 0.05 and ** *p* < 0.01 when compared with control group; # *p* < 0.05 and ## *p* < 0.01 when compared with the H_2_O_2_ inducing group.

As mentioned above, in the presence of H_2_O_2_ oxidative stress, the expression of COL1A1, COL2A1, and aggrecan (ACAN) genes were all downregulated. HA’s presence can scavenger the H_2_O_2_ oxidative stress-induced gene effect on chondrocytes, while the CeO_2_ cannot scavenger the H_2_O_2_ induced COL2A1 gene effect on chondrocytes. The presence of HA + CeO_2_ can scavenger the H_2_O_2_ oxidative stress on chondrocytes, and further enhance HA’s scavenger effect on the H_2_O_2_ oxidative stress induced COL1A1 and COL2A1 gene expression on chondrocytes (Figure 7).

### 2.7. Effect of CeO_2_ Nanoparticles-Loaded Hyaluronic Acid Treatment Glycosaminoglycan (GAG) Synthesis

**Figure 8:**
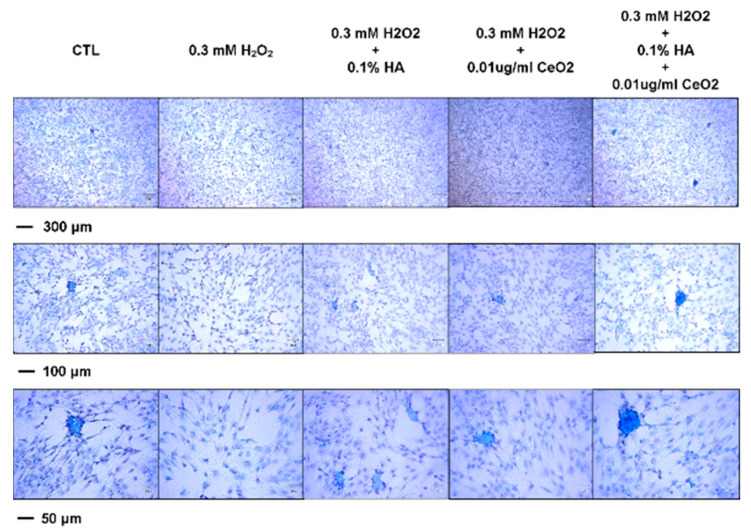
Effect of CeO_2_ nanoparticles-loaded hyaluronic acid treatment glycosaminoglycan (GAG) synthesis. Consistent with the gene expression patterns of ACAN, COL1A1, and COL2A1, H_2_O_2_ treatment can reduce the production of sulfated proteoglycan as determined by alcian blue staining. In the H_2_O_2_-treated samples, cellularity was relatively sparse with destructed cell membrane; whereas with the treatment of HA, CeO_2_ can reverse the H_2_O_2_-treatment effects. The accumulation of sulfated proteoglycan was most obvious when the cells were pretreated with both HA and CeO_2_.

Consistent with the gene expression patterns of ACAN, COL1A1, and COL2A1, H_2_O_2_ reduced production of sulfated proteoglycan as determined by alcian blue staining. In the H_2_O_2_-treated samples, cellularity was relatively sparse with destructed cell membrane as compared with the control samples. With the treatment of HA and CeO_2_, the effects of H_2_O_2_-treatment (both cellularity and cell membrane destruction) seem to be reversed. The accumulation of sulfated proteoglycan was recovered and was most obvious when the cells were pretreated with both HA and CeO_2_ (Figure 8).

## 3. Discussion

Osteoarthritis (OA) is a degenerative disease of articular cartilage induced by various factors. Although OA’s pathogenesis remains to be fully elucidated, it has been generally recognized that overexpression of reactive oxygen species (ROS) plays a vital role in the degeneration of articular cartilage [26]. Submillimolar concentrations of H_2_O_2_ can induce inhibition of the extracellular matrix (ECM) synthesis, chondrocyte apoptosis, lipid peroxidation, and inflammatory cytokines overproduction, and lead to the matrix metalloproteinase (MMPs) formation [5,6,7]. Effective disease-modifying OA therapies could lead to potentially transformative therapy. H_2_O_2_-induced oxidative stress helps study the occurrence and development of OA and evaluate the therapeutic strategies [27]. In this study, primary cultured chondrocytes treated with H_2_O_2_ to partly mimic their physiological conditions under oxidative stress were used as a model to examine the protective effects of cerium oxide and HA.

The particle size of synthesized cerium oxide nanoparticles was 131.1 ± 0.7 nm. Because the TEM images indicated that the grain size of CeO_2_ nanoparticles was around 10 nm, and the exposing surface was unstable, the larger nanoparticles may be due to the aggregation of the smaller ones [28]. H_2_O_2_ can be endogenously produced in OA’s pathogenesis and can induce chondrocytes injury [29]. This study found that inhibition of chondrocyte proliferation and chondrocytes-related gene expression was dose-dependent with H_2_O_2_ concentration. H_2_O_2_-mediated oxidative stress can enhance ROS and lipid peroxidation levels in chondrocytes. Previous studies have shown that chondrocyte apoptosis induced by oxidative stress was responsible for the development of OA [26,30,31]; lipid peroxidation could be induced by ROS and caused significant tissue damage in degenerative osteoarthritis [32,33,34,35].

Hyaluronic acid (HA), present in the healthy joint’s synovial fluid, has a protective effect against the invasion of PMN cells. In inflamed joints, the HA concentration decreases by depolymerization, the intraarticular application of high molecular weight HA might be an essential therapeutic regimen to restore the natural barrier against PMN migration and to interrupt the inflammatory cascade [36]. Cerium oxide nanoparticles, widely applied in our life [37], have recently come into consideration for biomedical use due to their potent antioxidant properties and have been proposed as a treatment for oxidative stress-associated chronic diseases [38,39,40]. Cerium oxide nanoparticles present the mimetic activity of superoxide dismutase. Mimicking natural antioxidant enzymes such as superoxide dismutase and catalase, the switching between CeO_2_ and CeO_2_-x during redox reactions makes CeO_2_ nanoparticles a lucrative catalytic nanoparticle. It is able to inactivate excess reactive oxygen species (ROS) which is correlated with a large number of pathologies [41]. CeO_2_ nanoparticles can scavenge most reactive oxygen species and nitrogen species via an auto-regenerative mechanism. In such circumstances, a minimum dose can exhibit catalytic activity for a longer duration [42].

Chondrocyte ECM mainly contains type 2 collagen and aggrecan. Chondrocyte apoptosis is closely related to the development and progression of osteoarthritis. A previous study has demonstrated that H_2_O_2_ can induce chondrocytes apoptosis and caspase-3 activation in rat chondrocytes [43]. In the study, both HA and CeO_2_ nanoparticles could lower chondrocytes apoptosis induced by H_2_O_2_. We also observed that COL2A1 and ACAN gene expression in chondrocytes was significantly decreased after H_2_O_2_ treatment. Both hyaluronic acid (HA) and CeO_2_ can scavenger H_2_O_2_ oxidative stress on chondrocytes, while the presence of HA + CeO_2_ can further enhance the scavenger effect of HA on the H_2_O_2_ oxidative stress induced COL1A1 and COL2A1 gene expression on chondrocytes. Inconsistent with the gene expression patterns of ACAN, COL1A1, and COL2A1, H_2_O_2_ treatment can significantly reduce the production of sulfated proteoglycan as determined by alcian blue staining; while the treatment of HA + CeO_2_ can reverse the H_2_O_2_-treatment effects. The accumulation of sulfated proteoglycan was most apparent when the cells were pretreated with both HA and CeO_2_. The results demonstrated that cartilage degeneration was significantly improved after CeO_2_ nanoparticles treatment and this tendency suggested that cerium oxide nanoparticles can protect damaged chondrocytes against oxidative stress. The results of this study indicate that cerium oxide nanoparticles can attenuate the progression of OA through suppression of H_2_O_2_-mediated injury.

## 4. Materials and Methods 

### 4.1. Materials

Cerium(III) nitrate hexahydrate (Ce(NO_3_)_3_·6H_2_O, Cat. No. 202991), hexamethylenetetramine (C_6_H_12_N_4_, Cat. No. 398160), sodium chloride (NaCl, Cat. No. S7653), sodium bicarbonate (NaHCO_3_, Cat. No. S5761), di-sodium hydrogen phosphate (Na_2_HPO_4_, Cat. No. 1.06585), and hyaluronic acid (HA, Cat. No. H7630) were obtained from Sigma-Aldrich (St. Louis, MO, USA).

### 4.2. Synthesize of Cerium Oxide Nanoparticles

CeO_2_ nanoparticles were synthesized according to the following manner [44]. In separate burettes, 0.02 M solution of cerium(III) nitrate hexahydrate was prepared by dissolving 2.17 g, Ce(NO_3_)_3_·6H_2_O in 250 mL distilled water and 0.03 M of K_2_CO_3_ solution was prepared by dissolving 1.036 g, K_2_CO_3_ in 250 mL distilled water. By adding drop by drop the aqueous solution of cerium(III) nitrate hexahydrate (50 mL) and potassium carbonate (20 mL) to a well stirred water (100 mL), a white precursor, cerium(III) carbonate, was precipitated. During the precipitation method, the constant Ph = 6 was maintained. The product was aged for 2.5 h (at 220 °C) without any washing and purification and finally calcined for 3 h (at 600 °C). The resulting CeO_2_ nanoparticles were dried for 2 h (at 65 °C), and then cooled to room temperature.

### 4.3. Characterization

#### 4.3.1. Morphology and Composition of Cerium Oxide Nanoparticles

Scanning electron microscopy (SEM) was used to examine the microstructure of the CeO_2_ nanoparticles. The specimens were mounted onto an adhesive copper stub, and then gold sputtered. SEM analyses were performed using a JSM-7600F (JEOL, Tokyo, Japan) electron microscope with a current and voltage of 20 mA and 10 kV, respectively. Additionally, electron dispersive spectrophotometry (EDS) were used to determine the composition of the synthesized particles. According to the Nanogenotox protocol [45], the 0.5% absolute ethanol pre-wetted CeO_2_ nanoparticles were dispersed at 2.56 mg/mL, in 0.05% bovine serum albumin (BSA), in double-distilled water by 16 min sonication. Then, the sonicated CeO_2_ nanoparticles were dispersed in DMEM high glucose with 10% FBS for the transmission electron microscopy (TEM) examination. A small drop of the stock suspension was pipetted onto a TEM grid and allowed to dry at room temperature and the observed by transmission electron microscopy (TEM) (JEM-2011, JEOL instrument, Tokyo, Japan) at a voltage of 200 kV.

#### 4.3.2. Particle Size Identification

At an incident angle of 90°, the particle sizes of the CeO_2_ nanoparticles were measured by dynamic light scattering (DLS) at 25 °C; while the zeta potential was determined by DLS associated with electrophoretic mobility at pH 7.4. Then, 5 mg CeO_2_ nanoparticles were dispersed in 5 mL de-ionized water for measurements of mean size. Then, the obtained dispersion was vortexed for 30 s with 5 repeats. After complete homogenization, the samples were placed in a cuvette for measurements in a Zeta-sizer Nano ZS (Malvern Instruments Ltd, Worcestershire, UK).

#### 4.3.3. Crystal Structure Identification

X-ray diffractometry (XRD, TTRAX III, Rigaku, TX, USA) was used to determine the crystal structure of the synthesized CeO_2_ nanoparticles [46]. By using a Ni filter with a potential of 30 kV and current of 15 mA, the synthesized CeO_2_ nanoparticles were collected and mounted onto the sample holder of the X-ray powder diffractometer under Cu KαI radiation (λ = 0.15406 nm). In the range from 20° to 80°, each specimen was scanned at a speed of 2°/min. The patterns were analyzed using a model auto-matched to the international center for the diffraction database using Jade 6.0 software. JCPDS Card No. 340394 was used as the standard pattern.

### 4.4. In Vitro Study

#### 4.4.1. Isolation of Chondrocytes

Cartilage from the knees of bovine was minced into small pieces, then sequentially digested in 0.25% trypsin for 30 min and placed on 2 mg/mL collagenase II-containing medium for 4–5 h, at 37 °C. The solution was washed using phosphate-buffered saline (PBS) and filtered through a 200 μm cell strainer. The cells were collected by centrifugation, and then cultured in DMEM/F12 medium containing 10% FBS in a humidified atmosphere (at 37 °C, 5% CO_2_). Chondrocytes at passage 2 were selected for the subsequent processes.

#### 4.4.2. Determination of Experimental Concentrations of H_2_O_2_

Chondrocytes exposed to H_2_O_2_ were used as the experimental oxidative stress model [25]. At the preparation of 1 × 10^5^/mL single-cell suspension, the cultured chondrocytes were seeded into 96-well plate at 10^4^ cells in each well. When chondrocytes adhered to the wall, the cells were starved for 24 h by adding 100 μL serum-free culture medium. Then, chondrocytes were treated with H_2_O_2_ (0, 0.1, 0.3, 1, 3, and 10 mM) for 30 min. Then, chondrocytes would be evaluated by lactate dehydrogenase (LDH) assays, water-soluble tetrazolium (WST-1) assays, and live/dead staining. Finally, cell apoptosis assay, gene expression, and alcian blue staining were used to evaluate the capacity of CeO_2_ nanoparticles and CeO_2_/HA.

#### 4.4.3. Cell Viability

The biocompatibility of the CeO_2_ nanoparticles was evaluated using the water-soluble tetrazolium (WST-1) assay (Sigma, St. Louis, MO, USA) [46]. The biocompatibility of the as-prepared composite was tested according to the ISO 10993-5 standard [47]. An extract medium was prepared by adding 0.2 g/mL of the CeO_2_ nanoparticles to high-glucose DMEM (Sigma, St. Louis, MO, USA), followed by incubation at 37 °C, for 24 h. At a cell density of 5 × 10^3^ cells/well, chondrocytes were seeded in 96-well plates and incubated at 37 °C, for 1 day. Then, the culture medium was replaced with the extract medium, and samples and cells were incubated for 1 to 3 days. Before the assay, 10 μL WST-1 reagent was added into each well for 4 h incubation; then, the plate was placed in a spectrophotometric plate reader (ELISA reader, Tecan Sunrise, Hombrechtikon, Switzerland) and read at the 450 nm absorbance (with a reference filter at 600 nm) to determine the amount of Formazan formed. The percentage of cell viability was calculated by the following Equation (1):

(1) $$\mathrm{Cell}\;\mathrm{viability}\;\left(\%\right)=\frac{\left(\mathrm{OD}\;\mathrm{experiment}\;-\;\mathrm{OD}\;\mathrm{background}\right)\;\times 100}{\left(\mathrm{OD}\;\mathrm{control}\;-\;\mathrm{OD}\;\mathrm{background}\right)}$$

#### 4.4.4. Cytotoxicity

The CytoTox 96 Assay Kit (Promega Corporation, Madison, WI, USA) for measuring the extracellular lactate dehydrogenase (LDH) content was used to evaluate the chondrocytes cytotoxicity. Briefly, after transferring the suspension medium to a new enzymatic assay plate, the LDH substrate solution was added for 30 min incubation; then, the stop solution was added, and the absorbance at 490 nm was measured on a spectrophotometric plate reader (ELISA reader, Tecan Sunrise, Hombrechtikon, Switzerland). The percentage of cytotoxicity was calculated by the following Equation (2):

(2) $$\mathrm{Cytotoxicity}\;\left(\%\right)=\frac{\left(\mathrm{Experimental}\;\mathrm{value}\;-\;\mathrm{Negative}\;\mathrm{control}\right)\;\times 100}{\left(\mathrm{Positive}\;\mathrm{control}-\;\mathrm{Negative}\;\mathrm{control}\right)}$$

#### 4.4.5. Live/Dead Assay

The Invitrogen LIVE/DEAD Viability/Cytotoxicity Kit containing approximately 2 μM calcein-AM and 4 μM EthD-1 as a working solution (Invitrogen/Thermo Fisher Scientific Inc., Waltham, MA, USA) was used to assess the cell viability of chondrocytes, according to the manufacturer’s instructions. Live cells are distinguished by producing an intense uniform green fluorescence in live cells (ex/em ~495 nm/~515 nm), while cells with damaged membranes produce a bright red fluorescence in dead cells (ex/em ~495 nm/~635 nm).

#### 4.4.6. Detection of Cell Apoptosis Rate by Flow Cytometry

The FITC Annexin V Apoptosis Detection Kit (Thermo Fisher Scientific Inc., MA 02451, USA) was used to quantify the percentage of cells undergoing apoptosis, according to the manufacturer’s instructions. Briefly, chondrocytes were harvested after treatment, washed twice with cold PBS, then re-suspended in 100 μL of binding buffer (containing 5 μL FITC Annexin V and 5 μL propidium iodide (PI)), incubated (at 25 °C) for 15 min in the dark), then 400 μL binding buffer was added, the cells were analyzed with a FACScan flow cytometer (BD Biosciences, San Jose, CA, USA).

#### 4.4.7. Gene Expression

The relative expression fold changes of three cartilage-related genes, including aggrecan (ACAN), collagen type 1 (COL1a1), and collagen type 2 (COL2a1), were quantified using real-time RT-PCR. The primers (Biotools Co., Ltd., Taipei, Taiwan) are shown in Table 2. For total RNA extraction, Qiazol (Qiagen, Valencia, CA, USA) was used, according to the manufacturer’s protocol. For the first-strand cDNA synthesis, random hexamers (Vivantis Inc., Oceanalde, CA, USA) and reverse transcriptase (Vivantis Cat No: RTPL12) were used with the following PCR parameters: 95 °C for denaturation (3 min), 40 cycles of 95 °C for 20 s, 60 °C for annealing (30 s), and 72 °C for elongation (30 s). TOOLS 2X SYBR qPCR Mix (Biotools Co., Ltd., Taipei, Taiwan) was applied for real-time RT-PCR using a CFX Connect Real-Time PCR Detection System (BioRed, CA, USA). The expression of the target genes was calculated by using glyceraldehyde 3-phosphate dehydrogenase (GAPDH) as an endogenous control.

#### 4.4.8. Alcian Blue Staining for Mucopolysaccharides

The deparaffinize slides were rehydrated with distilled water, stained with alcian blue solution (pH 2.5) for 30 min, washed by running tap water for 2 min, rinsed in distilled water, then counterstained with nuclear fast red solution, and mounted for later observation.

### 4.5. Statistical Analysis

All data were expressed as mean ± standard deviation (SD). Statistical analysis was performed by using one-way ANOVA and the post hoc comparisons used was Bonferroni test. Statistically significance was defined at p-value less than 0.05. All analyses were performed by using SPSS version 16.0 software (SPSS Inc., Chicago, IL, USA).

## 5. Conclusions

In this study, the particle size of synthesized cerium oxide nanoparticles was in the 131.1 ± 0.7 nm range, and the TEM images indicated that the grain size of CeO_2_ nanoparticles was around 10 nm. H_2_O_2_ can be endogenously produced in OA’s pathogenesis and can induce chondrocytes injury, and chondrocytes-related gene expression was dose-dependent with H_2_O_2_ concentration. Our findings suggest that CeO_2_ nanoparticles can prevent H_2_O_2_-induced chondrocytes injury through its antioxidant effects in vitro and reduced cartilage damage. Cerium oxide nanoparticles present the mimetic activity of superoxide dismutase. It was mimicking natural antioxidant enzymes such as superoxide dismutase and catalase, which could inactivate the excess of ROS correlated with a large number of pathologies. In vitro OA model, the results demonstrated that cartilage degeneration was significantly improved after CeO_2_ nanoparticles treatment and this tendency suggested that cerium oxide nanoparticles can protect damaged chondrocytes against oxidative stress. These results support the potential therapeutic applications of CeO_2_ nanoparticles as a supplementation in human OA treatment.

## Figures and Tables

**Table 1:** The percentage of each quadrant of figures of flow cytometry.

		Q1/%	Q2/%	Q3/%	Q4/%
a.	Normal cell	0	0	100	0
b.	Control group	1.7	2.9	94.8	0.6
c.	0.3 mM H_2_O_2_	3.1	3.3	93	0.6
d.	1 mM H_2_O_2_	7.4	26.9	29.6	36.2
e.	0.02 μg/mL CeO_2_	2.6	2.4	94.6	0.5
f.	1% HA	3.3	2.2	94.1	0.4
g.	0.02 μg/mL CeO_2_ + 0.3 mM H_2_O_2_	2.5	2.7	94.2	0.6
h.	1% HA + 0.3 mM H_2_O_2_	1.9	2.9	94.6	0.7
i.	0.02 μg/mL CeO_2_/1% HA + 0.3 mM H_2_O_2_	1.1	2.2	96	0.7

Q1, Annexin V-/PI+ (Q1) which identify cells with permeabilized membranes only; Q2, Annexin V+/PI+ (Q2) which characterize late stage apoptotic cells; Q3, Annexin V-/PI- (Q3) which represent living cell; Q4, Annexin V+/PI- (Q4) which represent early apoptotic cell; as unstained controls, normal cell used to gate for negative cell population.

**Table 2:** Primers used for gene expression.

Gene Name	Forward Primer (5′-3′)	Reverse Primer (5′-3′)
ACAN	AGATGGCACCCTCCGATAC	ACACACCTCGGAAGCAGAAG
COL 1A1	AGAGGTCGCCCTGGAGC	CAGGAACACCCTGTTCACCA
COL 2A1	GGAGGGAACGGTCCACGAT	AGTCCGCGTATCCACAA
GAPDH	GCATTGTGGAAGGGCTCA	GGGTAGGAACACGGAAGG

COL 1A1, Type I Collagen; COL 2A1, Type II Collagen; ACAN, Aggrecan; GAPDH, glyceraldehyde-3-phosphate dehydrogenase.

## References

[1] Huang Z.W., Zhou M., Wang Q., Zhu M.J., Chen S., Li H. (2017). Mechanical and hypoxia stress can cause chondrocytes apoptosis through over-activation of endoplasmic reticulum stress. Arch. Oral Biol..

[2] Helmick C.G., Felson D.T., Lawrence R.C., Gabriel S., Hirsch R., Kwoh C.K., Liang M.H., Kremers H.M., Mayes M.D., Merkel P.A. (2008). Estimates of the prevalence of arthritis and other rheumatic conditions in the United States: Part I. Arthritis Rheum-Us.

[3] Lawrence R.C., Felson D.T., Helmick C.G., Arnold L.M., Choi H., Deyo R.A., Gabriel S., Hirsch R., Hochberg M.C., Hunder G.G. (2008). Estimates of the prevalence of arthritis and other rheumatic conditions in the United States: Part II. Arthritis Rheum..

[4] Moskowitz R.W. (2009). The burden of osteoarthritis: Clinical and quality-of-life issues. Am. J. Manag. Care.

[5] Berenbaum F. (2013). Osteoarthritis as an inflammatory disease (osteoarthritis is not osteoarthrosis!). Osteoarthritis Cartilage.

[6] Ling S.M., Bathon J.M. (1998). Osteoarthritis in older adults. J. Am. Geriatr. Soc..

[7] Sperati A., Picconi O., Tancioni G., Agabiti N. (2008). Outcomes of hip replacement: A hospital-based longitudinal study in Lazio region (Italy). Ann. Ig..

[8] Lo G.H., LaValley M., McAlindon T., Felson D.T. (2003). Intra-articular hyaluronic acid in treatment of knee osteoarthritis: A meta-analysis. JAMA.

[9] Moreland L.W. (2003). Intra-articular hyaluronan (hyaluronic acid) and hylans for the treatment of osteoarthritis: Mechanisms of action. Arthritis Res. Ther..

[10] Goldberg V.M., Buckwalter J.A. (2005). Hyaluronans in the treatment of osteoarthritis of the knee: Evidence for disease-modifying activity. Osteoarthritis Cartilage.

[11] Babior B.M., Kipnes R.S., Curnutte J.T. (1973). Biological defense mechanisms. The production by leukocytes of superoxide, a potential bactericidal agent. J. Clin. Investig..

[12] Goldstein I.M., Roos D., Kaplan H.B., Weissmann G. (1975). Complement and immunoglobulins stimulate superoxide production by human leukocytes independently of phagocytosis. J. Clin. Investig..

[13] Johnston R.B., Lehmeyer J.E., Guthrie L.A. (1976). Generation of superoxide anion and chemiluminescence by human monocytes during phagocytosis and on contact with surface-bound immunoglobulin G. J. Exp. Med..

[14] McCord J.M. (1974). Free radicals and inflammation: Protection of synovial fluid by superoxide dismutase. Science.

[15] Greenwald R.A., Moy W.W., Lazarus D. (1976). Degradation of Cartilage Proteoglycans and Collagen by Superoxide Radical. Arthritis Rheum..

[16] Greenwald R.A., Moy W.W. (1979). Inhibition of collagen gelation by action of the superoxide radical. Arthritis Rheum..

[17] Esch F., Fabris S., Zhou L., Montini T., Africh C., Fornasiero P., Comelli G., Rosei R. (2005). Electron localization determines defect formation on ceria substrates. Science.

[18] Nelson B.C., Johnson M.E., Walker M.L., Riley K., Sims C.M. (2016). Antioxidant Cerium Oxide Nanoparticles in Biology and Medicine. Antioxidants (Basel).

[19] Karakoti A.S., Monteiro-Riviere N.A., Aggarwal R., Davis J.P., Self W.T., McGinnis J., Seal S. (2008). Nanoceria as antioxidant: Synthesis and biomedical applications. Jom.

[20] Reed K., Cormack A., Kulkarni A., Mayton M., Sayle D., Klaessig F., Stadler B. (2014). Exploring the properties and applications of nanoceria: Is there still plenty of room at the bottom?. Environ. Sci. Nano.

[21] Celardo I., Pedersen J.Z., Traversa E., Ghibelli L. (2011). Pharmacological potential of cerium oxide nanoparticles. Nanoscale.

[22] Narayanan K.B., Park H.H. (2013). Pleiotropic functions of antioxidant nanoparticles for longevity and medicine. Adv. Colloid Interface Sci..

[23] Walkey C., Das S., Seal S., Erlichman J., Heckman K., Ghibelli L., Traversa E., McGinnis J.F., Self W.T. (2015). Catalytic properties and biomedical applications of cerium oxide nanoparticles. Environ. Sci. Nano.

[24] Kwon H.J., Cha M.Y., Kim D., Kim D.K., Soh M., Shin K., Hyeon T., Mook-Jung I. (2016). Mitochondria-Targeting Ceria Nanoparticles as Antioxidants for Alzheimer’s Disease. Acs Nano.

[25] Zhuang C.P., Wang X.P., Chen T.S. (2013). H2O2 Induces Apoptosis of Rabbit Chondrocytes Via Both the Extrinsic and the Caspase-Independent Intrinsic Pathways. J. Innov. Opt. Health Sci..

[26] Pan Y.T., Chen D., Lu Q.Y., Liu L.F., Li X., Li Z.C. (2017). Baicalin prevents the apoptosis of endplate chondrocytes by inhibiting the oxidative stress induced by H2O2. Mol. Med. Rep..

[27] Liu-Bryan R., Terkeltaub R. (2015). Emerging regulators of the inflammatory process in osteoarthritis. Nat. Rev. Rheumatol..

[28] Cheung D.L. (2014). Aggregation of nanoparticles on one and two-component bilayer membranes. J. Chem. Phys..

[29] Li B., Jiang T.M., Liu H., Miao Z.K., Fang D.P., Zheng L., Zhao J.M. (2019). Andrographolide protects chondrocytes from oxidative stress injury by activation of the Keap1-Nrf2-Are signaling pathway J. Chem. Phys..

[30] Sun J., Wei X.L., Lu Y.D., Cui M., Li F.G., Lu J., Liu Y.J., Zhang X. (2017). Glutaredoxin 1 (GRX1) inhibits oxidative stress and apoptosis of chondrocytes by regulating CREB/HO-1 in osteoarthritis. Mol. Immunol..

[31] Sakata S., Hayashi S., Fujishiro T., Kawakita K., Kanzaki N., Hashimoto S., Lwasa K., Chinzei N., Kihara S., Haneda M. (2015). Oxidative Stress-induced Apoptosis and Matrix Loss of Chondrocytes Is Inhibited by Eicosapentaenoic Acid. J. Orthop. Res..

[32] Karakurum G., Karakok M., Tarakcioglu M., Kocer N.E., Kocabas R., Bagci C. (2003). Comparative effect of intra-articular administration of hyaluronan and/or cortisone with evaluation of malondialdehyde on degenerative osteoarthritis of the rabbit’s knee. Tohoku J. Exp. Med..

[33] Abusarah J., Bentz M., Benabdoune H., Rondon P.E., Shi Q., Fernandes J.C., Fahmi H., Benderdour M. (2017). An overview of the role of lipid peroxidation-derived 4-hydroxynonenal in osteoarthritis. Inflamm. Res..

[34] Aydogan N.H., Baydar M., Atay T., Perktas I., Baykal B., Ozmeric A. (2008). The effect of arthroscopic surgery and intraarticular drug injection to the antioxidation system and lipid peroxidation at osteoarthritis of knee. Saudi Med. J..

[35] Ostalowska A., Birkner E., Wiecha M.A., Kasperczyk S., Kasperczyk A., Kapolka D., Zon-Giebel A. (2006). Lipid peroxidation and antioxidant enzymes in synovial fluid of patients with primary and secondary osteoarthritis of the knee joint. Osteoarthritis Cartilage.

[36] Partsch G., Schwarzer C., Neumuller J., Dunky A., Petera P., Broll H., Ittner G., Jantsch S. (1989). Modulation of the Migration and Chemotaxis of Pmn Cells by Hyaluronic-Acid. Z. Rheumatol..

[37] Li Y., Li P., Yu H., Bian Y. (2016). Recent advances (2010–2015) in studies of cerium oxide nanoparticles’ health effects. Environ. Toxicol. Pharmacol..

[38] Rzigalinski B.A., Carfagna C.S., Ehrich M. (2017). Cerium oxide nanoparticles in neuroprotection and considerations for efficacy and safety. Wiley Interdiscip. Rev.-Nanomed. Nanobiotechnol..

[39] Cordoba-Jover B., Arce-Cerezo A., Ribera J., Pauta M., Oro D., Casals G., Fernandez-Varo G., Casals E., Puntes V., Jimenez W. (2019). Cerium oxide nanoparticles improve liver regeneration after acetaminophen-induced liver injury and partial hepatectomy in rats. J. Nanobiotechnol..

[40] Tatar T., Polat Y., Comu F.M., Kartal H., Arslan M., Kucku A. (2018). Effect of cerium oxide on erythrocyte deformability in rat lower extremity ischemia reperfusion injury. Bratisl. Med. J..

[41] Turin-Moleavin I.A., Fifere A., Lungoci A.-L., Rosca I., Coroaba A., Peptanariu D., Nastasa V., Pasca S.-A., Bostanaru A.-C., Mares M. (2019). In Vitro and In Vivo Antioxidant Activity of the New Magnetic-Cerium Oxide Nanoconjugates. Nanomaterials.

[42] Chen B.H., Inbaraj B.S. (2018). Various physicochemical and surface properties controlling the bioactivity of cerium oxide nanoparticles. Crit. Rev. Biotechnol..

[43] Hu J.Z., Cui W., Ding W., Gu Y., Wang Z., Fan W. (2017). Globular Adiponectin Attenuated H2O2-Induced Apoptosis in Rat Chondrocytes by Inducing Autophagy Through the AMPK/mTOR Pathway. Cell. Physiol. Biochem..

[44] Niu J.L., Azfer A., Rogers L.M., Wang X.H., Kolattukudy E.K. (2007). Cardioprotective effects of cerium oxide nanoparticles in a transgenic murine model of cardiomyopathy. Cardiovasc. Res..

[45] Rubio L., Marcos R., Hernandez A. (2018). Nanoceria acts as antioxidant in tumoral and transformed cells. Chem.-Biol. Interact..

[46] Fang C.H., Lin Y.W., Lin F.H., Sun J.S., Chao Y.H., Lin H.Y., Chang Z.C. (2019). Biomimetic Synthesis of Nanocrystalline Hydroxyapatite Composites: Therapeutic Potential and Effects on Bone Regeneration. Int. J. Mol. Sci..

[47] Wallin R.F., Arscott E. (1998). A practical guide to ISO 10993-5: Cytotoxicity. Med. Dev. Diagnostic Ind..

